# Stable disease after multimodal treatment of SMARCA4-deficient undifferentiated tumor concurrently involving the stomach and mediastinum: a case report

**DOI:** 10.3389/fonc.2026.1944183

**Published:** 2026-09-28

**Authors:** Jianbing Song, Yunzhi Xu, Jia Du, Hongyi Cai

**Affiliations:** 1 The First Clinical Medicine College of Gansu University of Traditional Chinese Medicine, Lanzhou, China; 2 Department of Radiotherapy I, Gansu Provincial People’s Hospital, Lanzhou, China

**Keywords:** BRG1, case report, gastric lesion, immunotherapy, mediastinal lesion, radiotherapy, SMARCA4 deficiency, undifferentiated tumor

## Abstract

We report a clinically rare SMARCA4-deficient undifferentiated tumor concurrently involving the stomach and mediastinum. A 54-year-old man presented with a 1-month history of left upper abdominal pain radiating to the anterior chest and back. Contrast-enhanced computed tomography of the chest and abdomen showed thickening and abnormal enhancement of the gastric wall along the greater curvature, multiple enlarged lymph nodes along the lesser curvature, and a posterior inferior mediastinal mass. Biopsies of both the gastric body lesion and the mediastinal mass revealed a poorly differentiated/undifferentiated malignant tumor, and additional immunohistochemistry supported SMARCA4/BRG1-deficient undifferentiated tumor at both sites. The similar undifferentiated morphology and loss of SMARCA4/BRG1 expression support a possible biological relationship between the two lesions but do not establish the primary site, direction of spread, or a shared clonal origin. Because paired molecular profiling, clonality analysis, and phylogenetic analysis were not performed, three possibilities cannot be reliably distinguished: a gastric primary with mediastinal metastasis; a mediastinal primary with gastric involvement or metastasis; and two independent primary tumors of the stomach and mediastinum. The symptomatic mediastinal lesion was treated with local radiotherapy using 6-MV X-rays and image-guided radiotherapy (IGRT), with prescribed doses of 67.2 Gy in 28 fractions to GTVboost, 61.6 Gy in 28 fractions to GTV, and 50.4 Gy in 28 fractions to PTV, together with systemic therapy. Computed tomography after the 25th fraction showed marked shrinkage of the mediastinal lesion, with substantial relief of left upper abdominal and radiating chest and back pain. The patient subsequently received radiotherapy to multiple lesions and several lines of systemic treatment and remained alive with stable disease at the last follow-up. This case highlights the uncertainty in determining the primary site and relationship between lesions when a SMARCA4-deficient undifferentiated tumor concurrently involves the stomach and mediastinum. In symptomatic patients with advanced disease, local radiotherapy combined with systemic treatment may contribute to pain relief and short-term disease control.

## 1 Introduction

SMARCA4 (SWI/SNF-related, matrix-associated, actin-dependent regulator of chromatin, subfamily A, member 4) is an important tumor-suppressor gene. Its protein product is a key transcriptional activator within the chromatin-remodeling complex required for DNA transcription (1). By regulating the wrapping and packaging of DNA around histones, SMARCA4 plays an important role in gene-expression control. SMARCA4 mutations commonly affect the highly conserved ATPase-dependent chromatin-remodeling domain of the encoded Brahma-related gene 1 (BRG1) protein (1). SMARCA4-deficient undifferentiated tumors have predominantly been reported in the thorax. In recent years, tumors at extrathoracic sites, including the stomach, have increasingly been recognized, although they remain exceedingly rare. Published series indicate that gastric SMARCA4-deficient undifferentiated carcinoma/tumor mainly occurs in middle-aged and older men, is often diagnosed at an advanced stage, and may show reduced or absent epithelial-marker expression, complete loss of BRG1 expression, and high proliferative activity (2–4). Clinical manifestations are nonspecific. Histologically, these tumors may be poorly differentiated or undifferentiated and may display rhabdoid features, without definite glandular differentiation. Their immunophenotype may also be atypical, leading to potential misclassification as conventional poorly differentiated gastric adenocarcinoma, neuroendocrine carcinoma, hematolymphoid neoplasm, germ-cell tumor, or a primary mediastinal malignancy (5). No standardized treatment has been established for gastric SMARCA4-deficient undifferentiated tumors; reported cases have generally received individualized chemotherapy, immunotherapy, or multimodal treatment (1–3, 6). Here, we report a patient with a SMARCA4-deficient undifferentiated tumor concurrently involving the stomach and mediastinum, with emphasis on the diagnostic uncertainty, biological features, combined radiotherapy and systemic treatment, and short-term outcome.

## 2 Patient information

A 54-year-old man presented to our hospital on 4 November 2025 with a 1-month history of left upper abdominal pain radiating to the anterior chest and back, with little relief after medication. No remarkable medical, personal, or family history was documented. Contrast-enhanced chest and abdominal CT showed thickening and abnormal enhancement of the gastric wall along the greater curvature, multiple enlarged lymph nodes along the lesser curvature, and a mediastinal mass measuring approximately 6.9 × 5.1 × 7.4 cm (**Figure 1**). Upper gastrointestinal endoscopy demonstrated a distinct lesion in the gastric body (**Figure 2**). The serum pro–gastrin-releasing peptide level was 63.70 pg/mL. Biopsy of the gastric body lesion showed a poorly differentiated/undifferentiated malignant tumor, initially considered possibly adenocarcinoma. Needle biopsy of the posterior inferior mediastinal mass likewise revealed a poorly differentiated/undifferentiated malignant tumor. Additional immunohistochemistry was performed to characterize both lesions and assess their possible origin. Both showed loss of SMARCA4/BRG1 expression with retained INI-1 (**Figure 3**). The available immunophenotype was negative for CK7, CK20, CDX2, Villin, CgA, CD56, CD20, CD3, CD45, PLAP, OCT3/4, AFP, and Glypican-3; SATB2 and CK8/18 were focally positive, Vimentin was partially positive, Syn was positive, the Ki-67 index was approximately 70%, SALL4 and SOX2 were positive, and p53 showed a missense mutation-type expression pattern. MSH2, MSH6, MLH1, and PMS2 expression was retained (**Figure 4**). No common KRAS mutation was detected. The morphology and available immunohistochemical profile supported a diagnosis of SMARCA4-deficient undifferentiated tumor at both sites and provided evidence against several epithelial, neuroendocrine, hematolymphoid, and germ-cell origins covered by the panel; however, entities requiring tests that were not performed—including EBER, NUT, CD30/ALK, and melanocytic markers—cannot be regarded as excluded. Following multidisciplinary review of the clinical, endoscopic, imaging, morphologic, and immunophenotypic findings, the case was conservatively described as a SMARCA4-deficient undifferentiated tumor concurrently involving the stomach and mediastinum, without designation of a definite primary site. At least three explanations remain possible: a gastric primary with mediastinal metastasis; a mediastinal primary with gastric involvement or metastasis; and two independent primary tumors arising in the stomach and mediastinum. Because paired molecular profiling, clonality analysis, and phylogenetic analysis were not performed, these possibilities cannot be reliably distinguished, and neither a shared clonal origin nor the direction of spread can be established.

**Figure 1:**
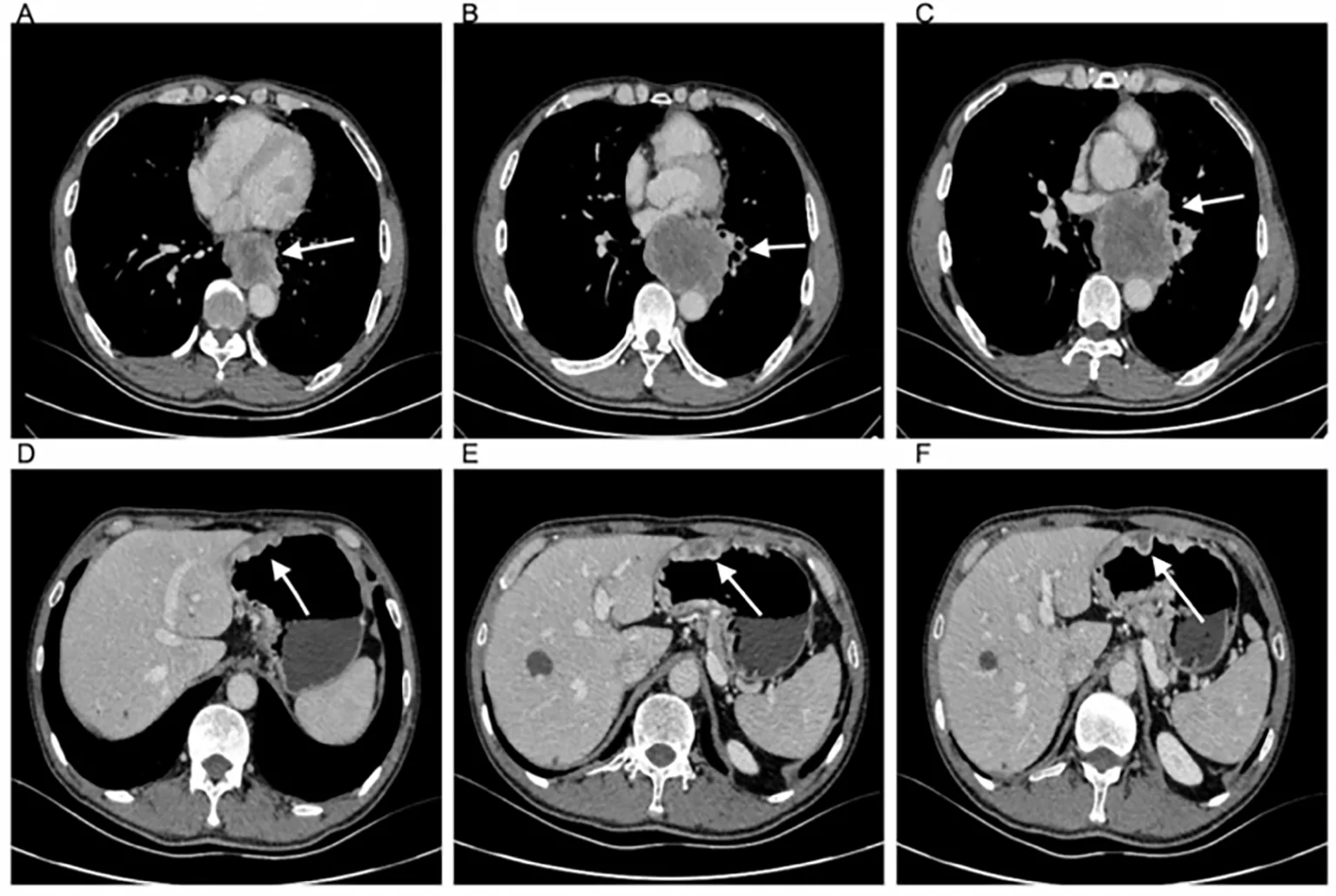
Contrast-enhanced computed tomography (CT) findings of the mediastinal and gastric lesions. **(A–C)** Axial contrast-enhanced chest CT images showing a mediastinal space-occupying lesion presenting as an irregular soft-tissue mass in the middle and lower mediastinum, closely associated with the adjacent esophagus and surrounding mediastinal structures, The arrow indicates the mediastinal lesion. **(D–F)** Axial contrast-enhanced abdominal CT images showing irregular wall thickening and an associated soft-tissue lesion along the greater curvature of the gastric body, with heterogeneous enhancement following contrast administration. The arrows indicate the gastric lesion.

**Figure 2:**
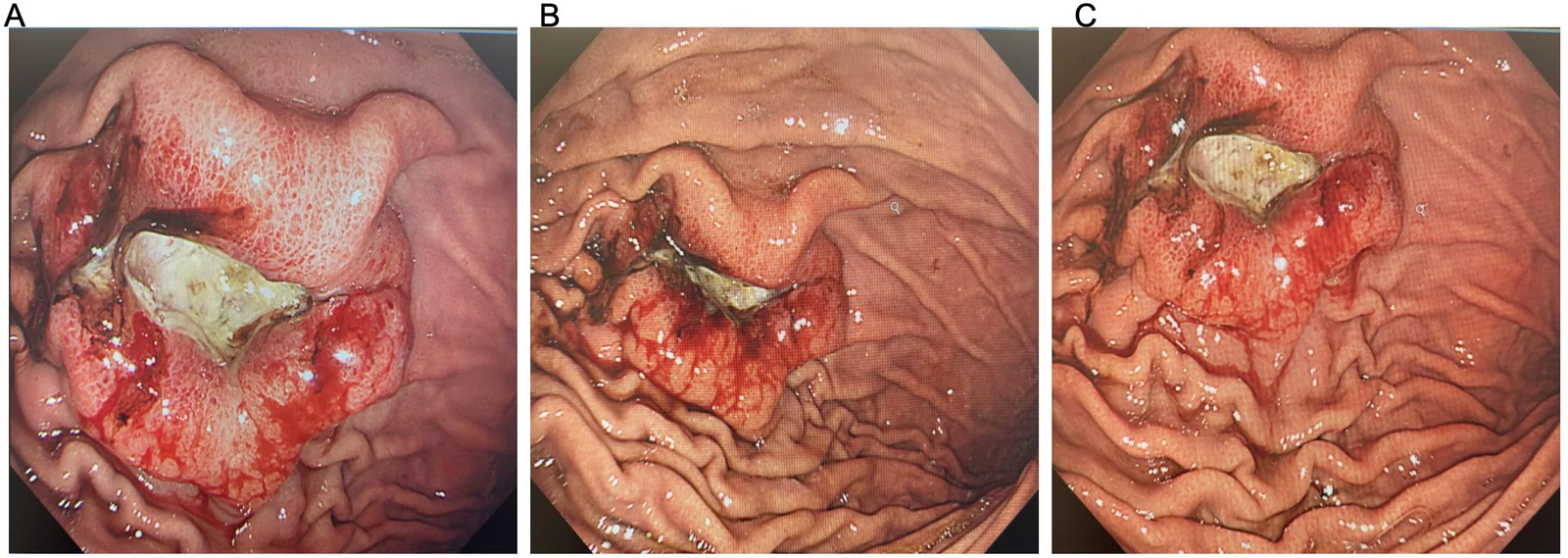
Endoscopic findings of the gastric lesion. **(A–C)** Gastroscopy revealed a large, irregular, elevated lesion along the greater curvature of the gastric body, characterized by a rough and hyperemic mucosal surface with prominent ulceration. The ulcer base was covered with yellowish-white exudate, with surrounding nodular mucosal elevation and hemorrhagic changes.

**Figure 3:**
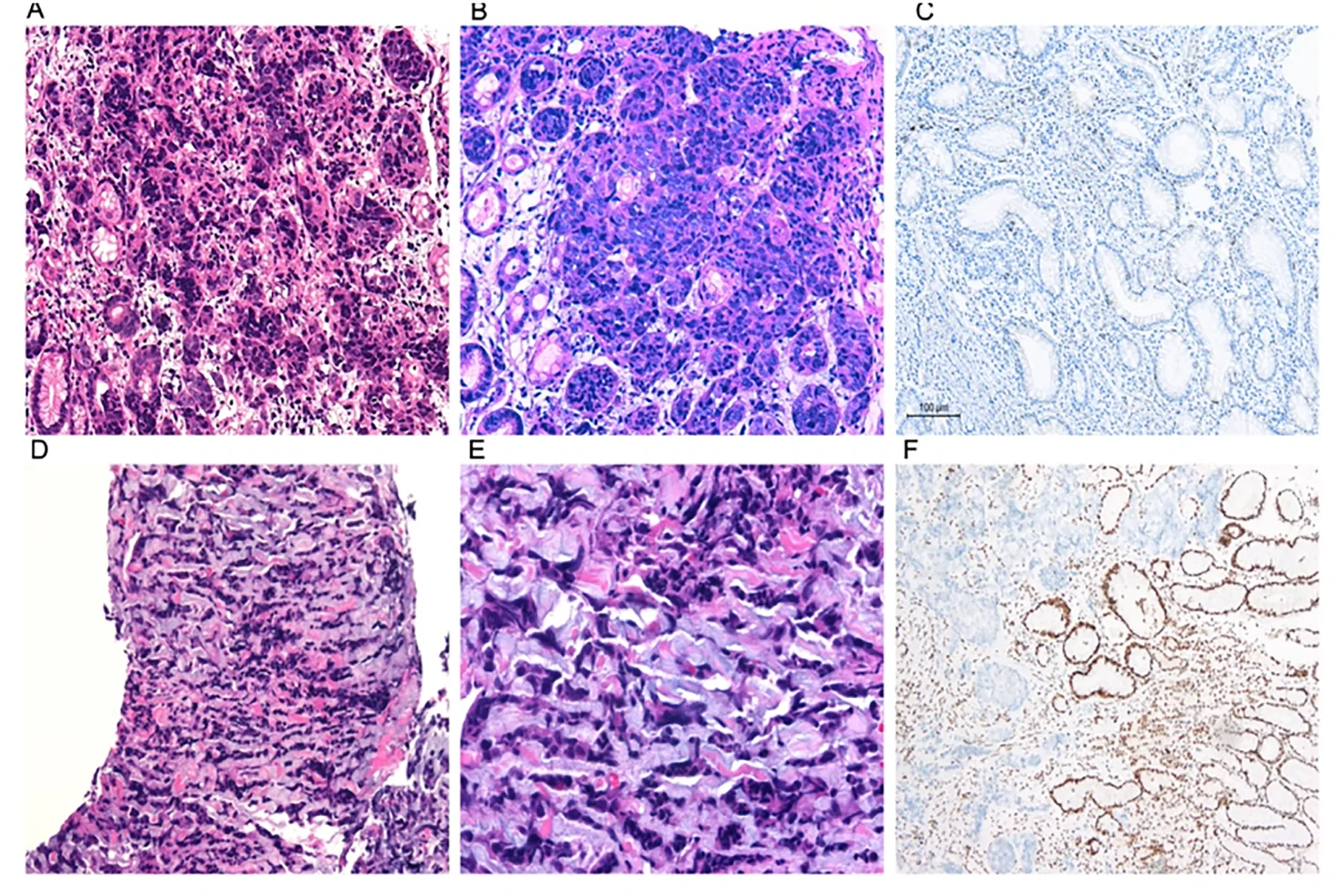
Histopathological and immunohistochemical features of the gastric and mediastinal lesions. **(A, B)** Hematoxylin and eosin (H&E) staining of the gastric lesion showing diffuse, solid growth of markedly atypical tumor cells with hyperchromatic and irregular nuclei. **(C)** Immunohistochemical staining of the gastric lesion showing loss of nuclear SMARCA4 (BRG1) expression in tumor cells, with retained nuclear expression in the surrounding non-neoplastic cells. **(D, E)** H&E staining of the mediastinal lesion showing infiltrative growth of atypical tumor cells, with histomorphological features similar to those of the gastric lesion. **(F)** Immunohistochemical staining of the mediastinal lesion showing loss of nuclear SMARCA4 (BRG1) expression in tumor cells, with retained nuclear expression in the surrounding non-neoplastic cells.

**Figure 4:**
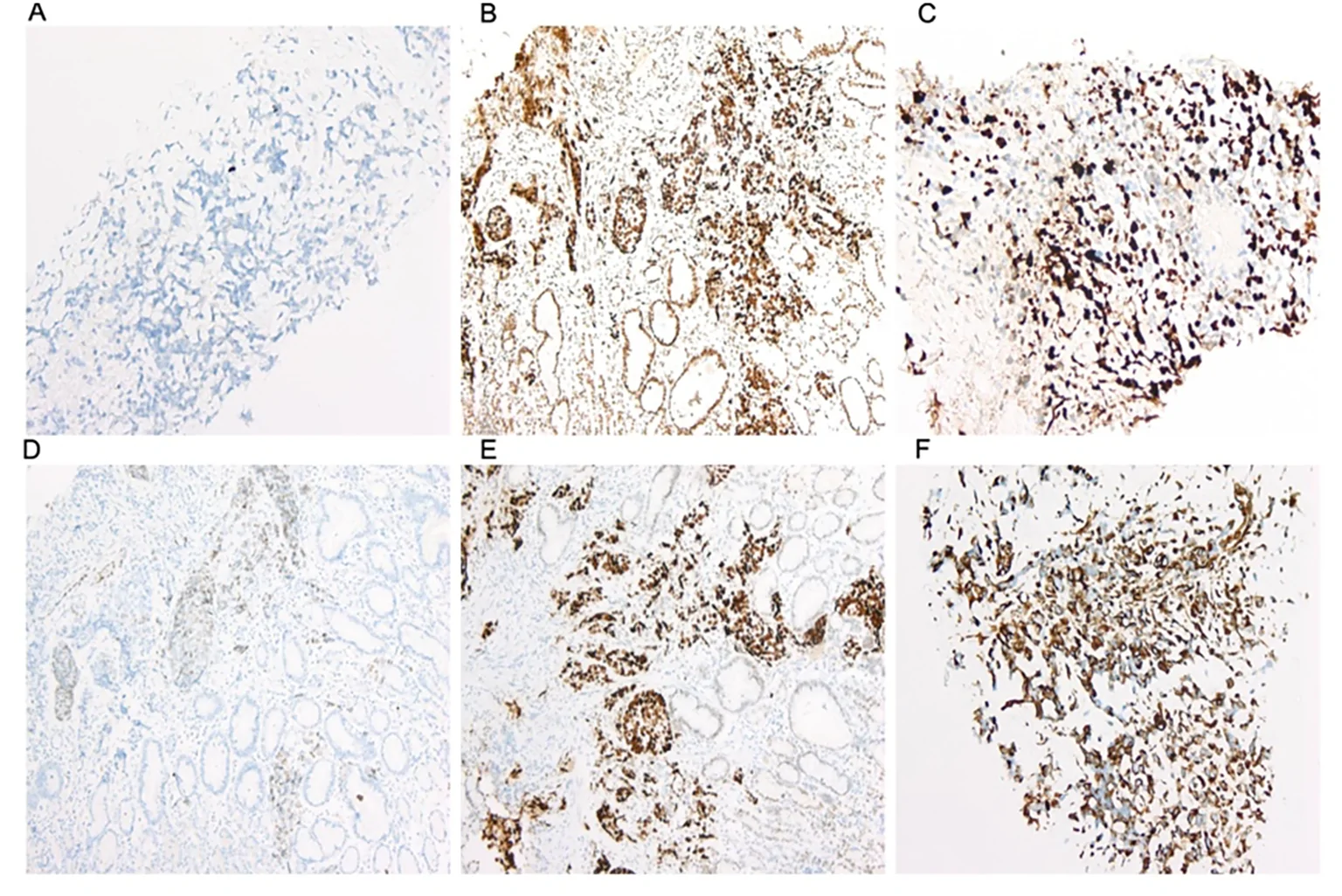
Immunohistochemical features of the mediastinal lesion. **(A)** CK8/18 showing focal positive expression. **(B)** Retained nuclear expression of INI-1. **(C)** Ki-67 proliferation index of approximately 70% in hotspot areas, indicating high proliferative activity. **(D)** Positive expression of SALL4. **(E)** Positive expression of SOX2. **(F)** Partial positive expression of vimentin.

After contraindications to radiotherapy had been excluded during the first hospitalization, local radiotherapy was delivered to the mediastinal lesion. The radiotherapy plan was as follows (1): Simulation and positioning: the patient was positioned supine and immobilized with a thermoplastic shell. CT simulation was performed with a 5-mm slice thickness (2). Gross tumor volume definition: the gross tumor volume (GTV) encompassed the radiologically visible mediastinal mass; GTVboost was generated by a 2-cm contraction from the GTV; and the planning target volume (PTV) was generated by a uniform 3-mm expansion of the GTV (**Figures 5**, **6**). No separate clinical target volume was defined, and no additional respiratory-gating or breath-hold technique was used (3). Treatment technique: treatment was delivered with a 6-MV linear accelerator and image-guided radiotherapy (IGRT). Prescribed doses were GTVboost 67.2 Gy in 28 fractions (2.4 Gy/fraction), GTV 61.6 Gy in 28 fractions (2.2 Gy/fraction), and PTV 50.4 Gy in 28 fractions (1.8 Gy/fraction) (4). Planned organ-at-risk constraints were spinal cord Dmax ≤45 Gy; for each lung, V20 ≤25%, V30 ≤18%, V5 ≤50%, and mean dose ≤13 Gy; heart V30 ≤40% and V40 ≤30%; and esophagus Dmax ≤63 Gy (5). Dose distribution: the thoracic IGRT plan achieved a highly conformal dose distribution, with 95% of the PTV receiving the prescribed dose while limiting irradiation of surrounding normal tissues. S-1 plus tislelizumab was administered concurrently for four cycles, with oxaliplatin added in cycle 1. Chest CT after 25 fractions showed marked shrinkage of the posterior inferior mediastinal lesion (**Figure 7**), and the abdominal pain and radiating chest and back pain were substantially relieved. No clinically evident radiation esophagitis or significant radiation dermatitis was observed during the first course of radiotherapy. Malnutrition, pulmonary infection, myelosuppression, pain, sleep disturbance, and mild nausea occurred during multimodal treatment and improved with fluid replacement, anti-infective therapy, analgesia, nutritional support, and leukocyte-stimulating treatment. During the second hospitalization, because multiple lesions were present throughout the body, the patient received VMAT to multiple sites using five PTVs: PTV1, a left apical paravertebral pleural lesion, 45 Gy in 15 fractions (3.0 Gy/fraction); PTV2, a right axillary lesion, 45 Gy in 15 fractions (3.0 Gy/fraction); PTV3, lesions around the left hilum and descending aorta, 22.5 Gy in 15 fractions (1.5 Gy/fraction); PTV4, a left apical costopleural lesion, 51 Gy in 15 fractions (3.4 Gy/fraction); and PTV5, a lesion along the greater curvature of the gastric body, 45 Gy in 15 fractions (3.0 Gy/fraction) (**Figure 8**). Planned organ-at-risk constraints were spinal cord Dmax ≤33 Gy, with Dmax ≤16 Gy for the cord adjacent to PTV3; for each lung, V20 ≤10% and V30 ≤5%; heart V20 ≤10%; esophagus and trachea Dmax ≤23 Gy; liver V5 ≤5%; and stomach Dmax ≤49 Gy and mean dose ≤30 Gy. Oxaliplatin, tislelizumab, and S-1 were administered concurrently. Subsequent admissions were for systemic treatment. At the last follow-up on 1 April 2026, the patient was alive with stable disease.

**Figure 5:**
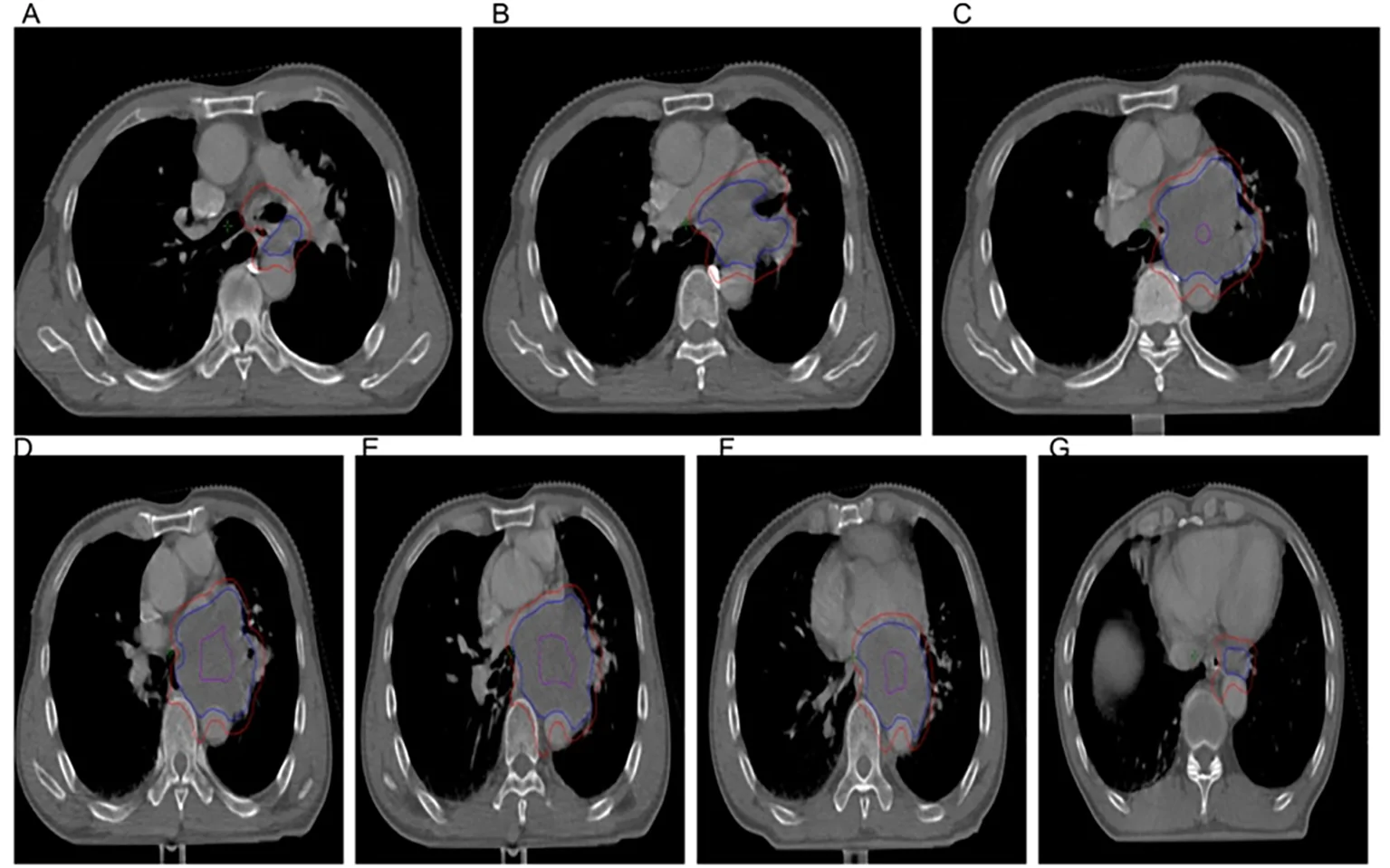
Delineation of the radiotherapy target volumes for the mediastinal lesion. **(A–G)** Axial CT images at different levels showing the delineation of the radiotherapy target volumes for the mediastinal lesion. The purple contour represents the gross tumor volume for boost irradiation (GTVboost), the blue contour represents the gross tumor volume (GTV), and the red contour represents the planning target volume (PTV).

**Figure 6:**
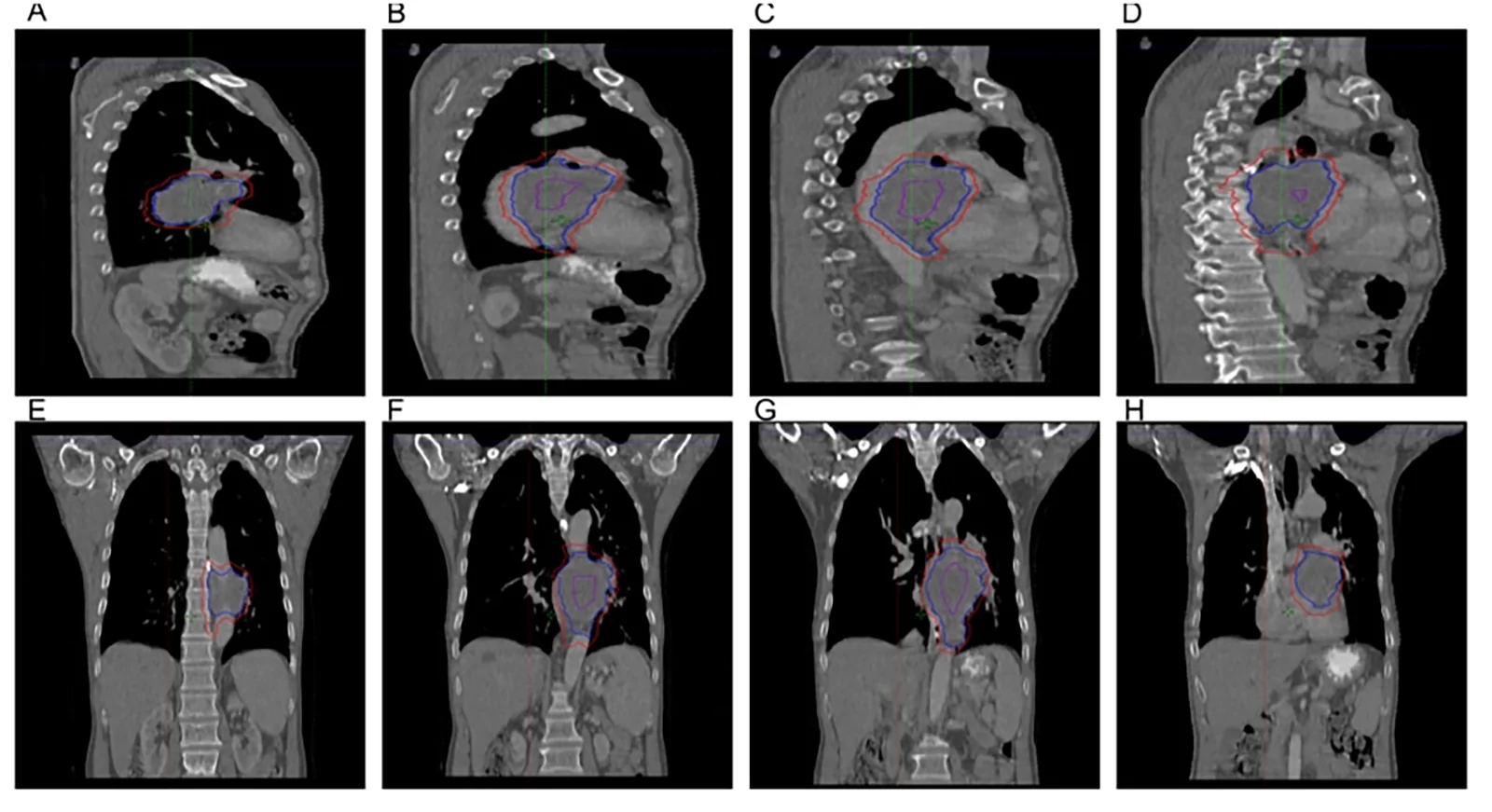
Delineation of the radiotherapy target volumes for the mediastinal lesion. **(A–D)** Sagittal CT images at different levels showing the delineation of the radiotherapy target volumes for the mediastinal lesion; **(E–H)** coronal CT images at different levels showing the delineation of the radiotherapy target volumes. The purple contour represents the gross tumor volume for boost irradiation (GTVboost), the blue contour represents the gross tumor volume (GTV), and the red contour represents the planning target volume (PTV).

**Figure 7:**
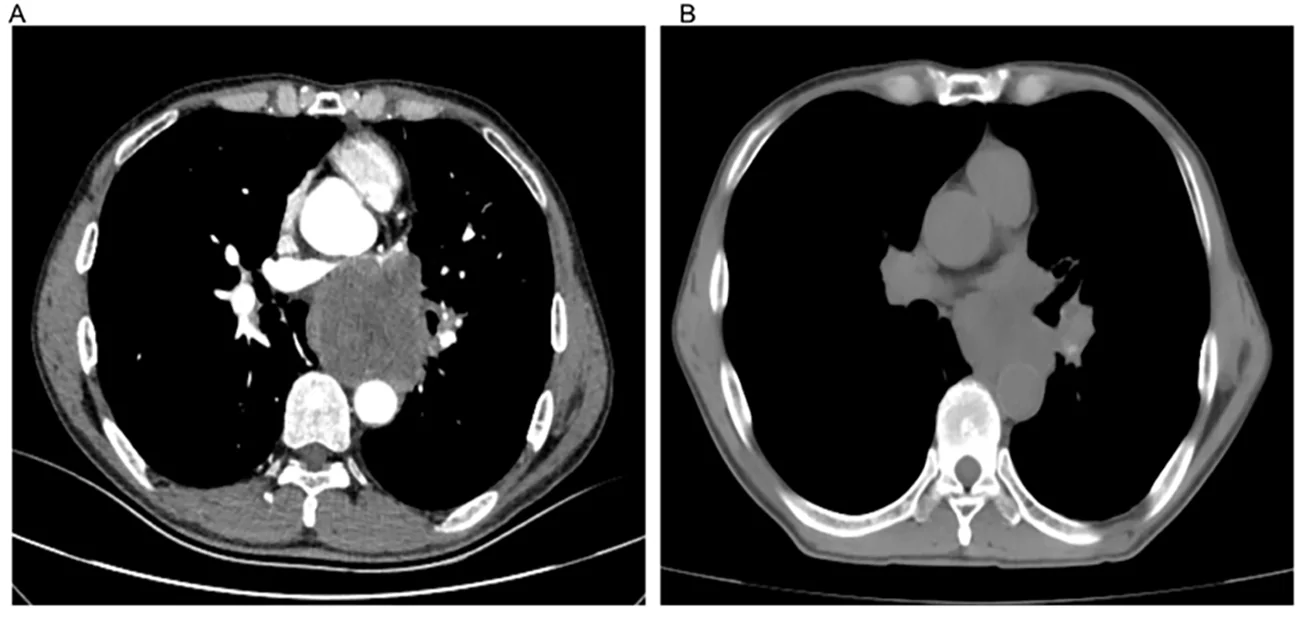
Comparison of chest computed tomography findings before and after treatment. **(A)** Pretreatment contrast-enhanced chest computed tomography shows a large irregular soft-tissue mass in the mediastinum, predominantly involving the left mediastinal and hilar regions. The lesion is closely associated with the adjacent great vessels, bronchi and surrounding mediastinal structures and causes substantial compression of the neighboring tissues. **(B)** Post-treatment chest computed tomography demonstrates a marked reduction in the size of the mediastinal mass and alleviation of compression on the adjacent structures, indicating an imaging response to treatment.

**Figure 8:**
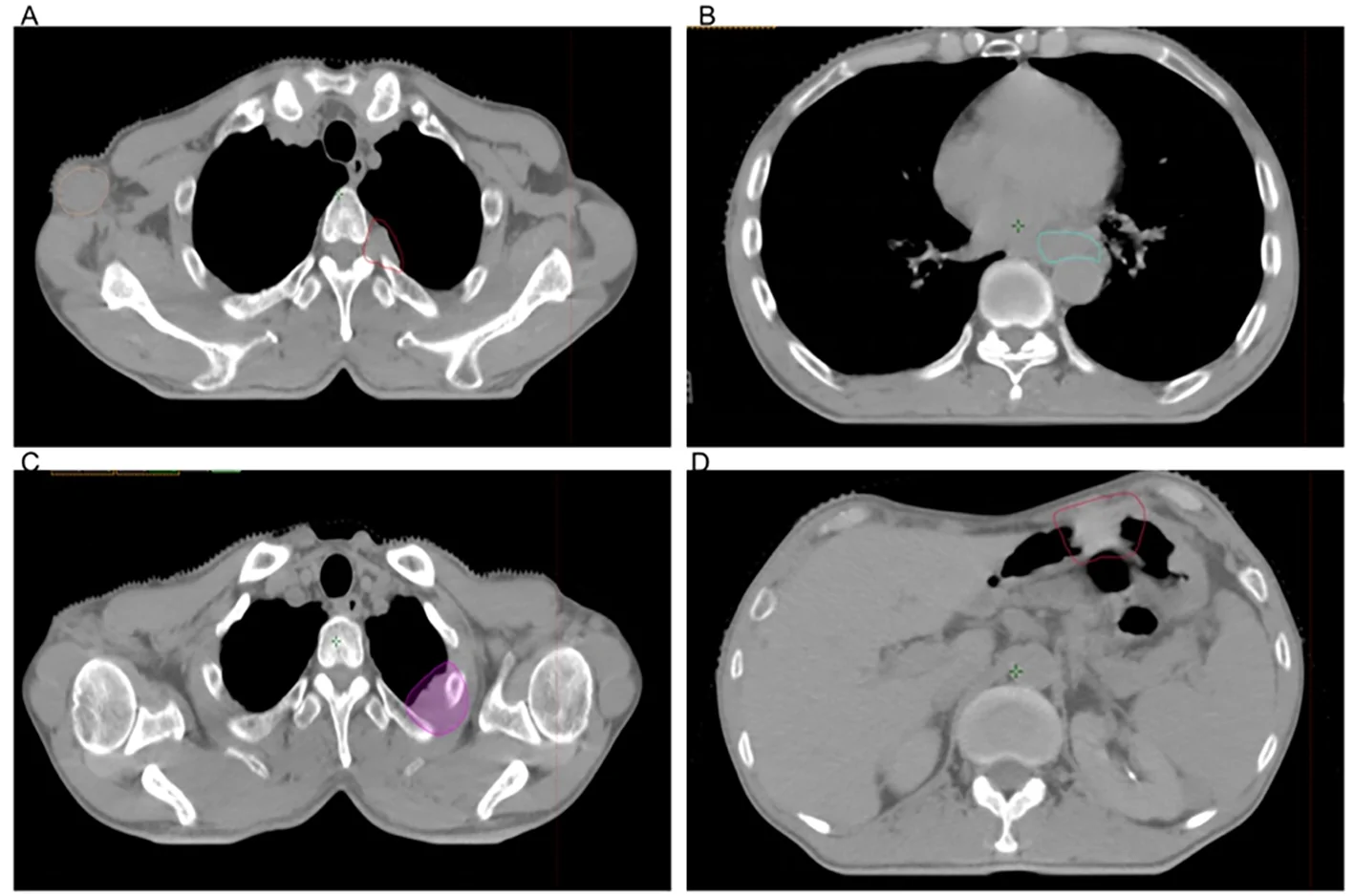
Delineation of radiotherapy target volumes for multiple lesions during the second hospitalization. **(A)** PTV1 encompasses the left apical paravertebral pleural lesion, while PTV2 encompasses the right axillary lesion. **(B)** PTV3 encompasses the lesions in the left hilar and peridescending aortic regions. **(C)** PTV4 encompasses the left apical intercostal pleural lesion. **(D)** PTV5 encompasses the lesion along the greater curvature of the gastric body.

## 3 Discussion

Primary gastric SMARCA4-deficient undifferentiated tumors are exceedingly rare, and current knowledge is derived mainly from case reports and small case series. Available reports indicate a predominance in middle-aged and older men, nonspecific clinical manifestations, and presentation with abdominal pain, gastrointestinal bleeding, anemia, or other upper gastrointestinal symptoms; some patients already have nodal or distant disease at diagnosis (2–4, 6–8). Histologically, these tumors typically exhibit high-grade poorly differentiated or undifferentiated morphology, with sheet-like, nested, or diffuse solid growth. Some show rhabdoid features, whereas the present case lacked definite glandular differentiation. Complete loss of nuclear BRG1/SMARCA4 expression in tumor cells is a key immunophenotypic feature. Epithelial markers may be only focally expressed or entirely absent, whereas INI1/SMARCB1 is generally retained (1–4, 7, 8). Because of morphologic and immunophenotypic heterogeneity, small biopsy specimens may initially be diagnosed as poorly differentiated carcinoma or an undifferentiated malignant tumor. SMARCA4 is located at chromosome 19p13.2 and encodes BRG1, a core ATPase subunit of the SWI/SNF chromatin-remodeling complex. This complex regulates chromatin accessibility in an ATP-dependent manner and participates in transcriptional control and cellular differentiation (1, 5). Pathogenic SMARCA4 alterations have been identified in some reported gastric cases, but the limited number of cases has precluded definition of a consistent, specific co-mutation profile (2, 4, 6). Diagnosis relies primarily on morphology and immunohistochemistry, and BRG1 staining is one of the most direct pathological clues. Molecular testing can provide additional evidence of SMARCA4 abnormalities. In poorly differentiated or undifferentiated gastric malignancies, particularly when conventional epithelial markers are reduced or absent, supplementary BRG1/SMARCA4 testing should be considered (1, 2, 4). Loss of BRG1 should consist of complete absence of nuclear staining in tumor cells, with retained nuclear staining in stromal, inflammatory, or other non-neoplastic cells as an internal control. Further differential diagnosis requires integration of cytokeratins, Vimentin, neuroendocrine markers, hematolymphoid markers, germ-cell tumor markers, and INI1 to assess conventional poorly differentiated gastric adenocarcinoma, neuroendocrine carcinoma, lymphoma, germ-cell tumor, and other SWI/SNF-complex-deficient tumors (1, 4, 8). In this case, both the gastric body lesion and posterior inferior mediastinal lesion were poorly differentiated/undifferentiated malignant tumors with loss of SMARCA4/BRG1 and retained INI1. Negative hematolymphoid and germ-cell markers, together with the epithelial- and neuroendocrine-marker expression pattern, informed the differential diagnosis, although the panel was incomplete. In particular, EBER, NUT, CD30/ALK, and melanocytic markers were not examined; therefore, diseases requiring these tests cannot be considered definitively excluded.

The principal diagnostic issue is the relationship between the gastric and mediastinal lesions. On reassessment, at least three explanations remain (1): a gastric primary with mediastinal metastasis (2); a mediastinal primary with gastric involvement or metastasis; and (3) two independent primary tumors of the stomach and mediastinum. The mediastinal mass was substantially larger than the gastric lesion, and neither lesion size nor the available imaging findings can reliably establish the stomach as the primary site. Although upper gastrointestinal endoscopy demonstrated a distinct gastric body lesion, SMARCA4-deficient undifferentiated tumors are more commonly encountered in the thorax, making a mediastinal primary with gastric involvement an important possibility. Conversely, the rarity of both lesions and their similar undifferentiated morphology and loss of SMARCA4/BRG1 support a possible biological relationship, but these features neither identify the primary site nor completely exclude two independent primary tumors. Crucially, paired molecular profiling, clonality analysis, and phylogenetic analysis were not performed. The available evidence therefore cannot establish a shared clonal origin or the direction of dissemination, and the three possibilities cannot be reliably distinguished. In light of these limitations, this case is best described conservatively as a SMARCA4-deficient undifferentiated tumor concurrently involving the stomach and mediastinum, without overinterpretation of the primary site or interlesional relationship.

Because few cases have been reported, no standard treatment has been established. Reported approaches include surgery, platinum-containing chemotherapy, fluoropyrimidine-based regimens, immunotherapy, and multimodal treatment. For localized disease, surgery can provide diagnostic tissue and local control, although some patients develop early recurrence or distant disease after resection (2, 4, 7). In advanced disease, treatment is individualized according to performance status and disease burden, often drawing on gastric-cancer regimens, but responses vary substantially (1–3, 6). Pan-cancer studies suggest that SMARCA4 alterations may correlate with immune infiltration and prognosis; however, these associations are tumor-type dependent, and SMARCA4 deficiency alone is not a validated predictive biomarker for immunotherapy (9). Disease control with a PD-1 antibody plus chemotherapy has been reported in SMARCA4-deficient undifferentiated carcinoma of the gastrointestinal tract (10), and some gastric cases have received chemotherapy combined with immune-checkpoint inhibitors, although the evidence remains limited to case reports and small retrospective series. SMARCA4/BRG1 is a core ATPase subunit of the SWI/SNF chromatin-remodeling complex. Its loss can disrupt chromatin regulation and may create targetable epigenetic dependencies. Functional antagonism exists between SWI/SNF-complex deficiency and polycomb repressive complex 2 (PRC2)-mediated epigenetic regulation, and EZH2, the catalytic subunit of PRC2, has emerged as a potential target in SWI/SNF-deficient tumors. Preclinical studies have shown sensitivity to EZH2 inhibition in some SMARCA4-mutant tumor models, with cellular senescence, apoptosis, and suppression of *in vivo* tumor growth; this sensitivity may be associated with low SMARCA2 expression (11). Nevertheless, direct clinical evidence for EZH2 inhibitors in this rare tumor remains lacking. EZH2-directed therapy should therefore be regarded as a biologically plausible research direction rather than an established strategy, and clinical studies are needed to clarify efficacy and predictive biomarkers. Reports of radiotherapy for SMARCA4-deficient undifferentiated tumors involving the stomach and mediastinum are particularly limited. No dedicated studies have defined radiosensitivity, optimal dose, or the benefit of combining radiotherapy with immunotherapy. In the present case, the posterior inferior mediastinal lesion was bulky and caused pronounced radiating chest and back pain; local IGRT was therefore delivered to the symptom-dominant lesion. Imaging during treatment showed marked tumor shrinkage, accompanied by substantial pain relief. This case suggests that, in advanced SMARCA4-deficient undifferentiated tumors, local radiotherapy to a bulky symptomatic lesion may be considered as an individualized approach alongside systemic treatment.

## 4 Conclusion

SMARCA4-deficient undifferentiated tumors concurrently involving the stomach and mediastinum pose a substantial diagnostic challenge. A gastric primary with mediastinal metastasis, a mediastinal primary with gastric involvement or metastasis, and two independent primary tumors of the stomach and mediastinum are all plausible explanations. The similar undifferentiated morphology and loss of SMARCA4/BRG1 at both sites support only a possible biological relationship. In the absence of paired molecular profiling, clonality analysis, or phylogenetic analysis, the primary site, direction of dissemination, and shared clonal origin cannot be reliably determined. The clinical course suggests that local radiotherapy combined with individualized systemic treatment may relieve pain and provide short-term disease control in selected patients; however, the optimal treatment and long-term prognosis require validation in additional cases and prospective studies.

## Data Availability

The original contributions presented in the study are included in the article/supplementary material, further inquiries can be directed to the corresponding author/s.
